# Exploring phenotypic diversity and constructing a floral core collection of *Ziziphus jujuba* var. *spinosa*

**DOI:** 10.7717/peerj.21708

**Published:** 2026-09-24

**Authors:** Yuyu Wang, Yuncheng Zhang, Yongqiang Sun, Kaiyun Wang, Xin Zhao, Jianhua Chen, Shengjun Dong

**Affiliations:** 1 College of Forestry, Shenyang Agricultural University, Shenyang, China; 2 Key Laboratory for Silviculture of Liaoning Province, Shenyang Agricultural University, Shenyang, China; 3 Forestry and Grassland Bureau of Karaqin Left Wing Mongolian Autonomous County, Kazuo, China

**Keywords:** *Ziziphus jujuba* var. *spinosa*, Floral organ traits, PCA, Environmental factors, Core collection

## Abstract

**Background:**

*Ziziphus jujuba* var. *spinosa* is an ecologically valuable tree species. However, most investigations have concentrated on the leaves and fruits. There is insufficient focus on investigating the phenotypic diversity and morphological characteristics of wild jujube floral organs. This gap in knowledge impedes a comprehensive understanding of floral development, which in turn restricts practical applications such as the enhancement of breeding efficiency and yield in cultivated varieties.

**Methods:**

We evaluated 28 floral traits of 205 sour jujube clones from 30 provenances across eight Chinese provinces. Phenotypic variation was assessed using nested ANOVA, coefficient of variation (CV), and Shannon-Wiener index (H′). Pearson correlation analysis was conducted to examine associations between floral traits and climatic and geographic factors. Principal component analysis (PCA) and hierarchical clustering were used to classify breeding groups, and a core collection was established *via* stepwise clustering.

**Results:**

The results demonstrated significant diversity and variation in the floral organs of sour jujube. The average CV for quantitative and qualitative traits were 25.30% and 37.85%, respectively, while the mean H′ for quantitative traits (1.68) was higher than that for qualitative traits (0.81). Variation among provenances was the primary source of phenotypic diversity, with the mean phenotypic differentiation coefficient for the quantitative traits of sour jujube reaching 71.32%. Size-related floral traits, including flower diameter, flower area, and flower disc diameter, were significantly correlated with temperature. These traits exhibited significant negative correlations with mean annual, January, and July temperatures (*P* < 0.01). Principal component analysis extracted six principal components, collectively accounting for 73.14% of the total variance. Based on phenotypic traits, the 205 clones were classified into five breeding groups: A and B for small- and large-flowered ornamental varieties, C for high-yield potential, and E for nectar-rich types with large disc areas. A specialized core collection of 51 accessions for the study of floral organs was constructed using a stepwise clustering approach. This work provides valuable insights for the evaluation and utilization of sour jujube germplasm.

## Introduction

Sour jujube (*Ziziphus jujuba* var. *spinosa*) belongs to the family Rhamnaceae, genus *Ziziphus* Mill., and is native to China (Zhu et al., 2023). Its distribution encompasses the tropical and subtropical regions of Asia and the Americas, as well as the hilly and mountainous areas of northern China (Liu & Liu, 2023). This species predominantly occurs under wild or semi-wild conditions, and often forms dense clusters or scattered distributions. Sour jujube is notable for its ability to grow in poor, arid, and saline-alkali soils and is considered highly tolerant of such conditions. Sour jujube trees play important ecological roles in wind-breaking, sand fixation, and soil conservation. Furthermore, sour jujube is rich in nutrients, making it an important tonic and medicinal material (Li et al., 2024).

Studies on plant phenotypic diversity are a fundamental approach for identifying superior resources and effectively utilizing existing germplasms (Yan et al., 2024; Vergara et al., 2021). Wild sour jujube populations exhibit a highly heterozygous background and substantial phenotypic variation. Several studies have investigated the phenotypic diversity of *Ziziphus* species, including *Z. jujuba* var. *spinosa* (Zhang et al., 2015), *Z. jujuba* Mill. (Khadivi et al., 2021), and *Z. mauritiana* Lam. (Sharif et al., 2019). Despite extensive studies on leaves and fruits, floral organs of wild sour jujube remain poorly characterized, resulting in a fundamental lack of understanding regarding the geographical variation patterns, adaptive significance, and potential for core collection development related to floral traits in sour jujube.

The flower organs of sour jujubes play pivotal roles in facilitating sexual reproduction and preserving phenotypic diversity. Its variations are of significant adaptive and evolutionary value, thereby forming a fundamental basis for phenotypic diversity (Pavlin et al., 2023; Zhou et al., 2019). Floral traits are not only key indicators of reproductive success and pollination biology (Harder & Johnson, 2009) but also have direct practical implications for breeding, cultivation, and resource management. For instance, flower size, morphology, and phenology can influence pollination efficiency, fruit set, and ultimately yield stability (Khadivi, 2018). Variations in floral architecture may be associated with local pollinators or environmental conditions, offering insights for selecting accessions suited to specific agro-ecological settings (Huang, Sun & Takahashi, 2022; Junker et al., 2013). Additionally, floral characteristics can serve as early selection markers in breeding programs, aiding in the identification of desirable traits linked to productivity, stress tolerance, or fruit quality before fruit development (Bassi, Negri & Magnani, 2021; De Oliveira, Dias & Dantas, 2011).

Significant differences in the quantity, type, size, shape, color, and arrangement of floral organs determine their dimensional and complex morphological characteristics (Endress, 2011; Wessinger & Hileman, 2016), which may be influenced by both genetic and environmental factors (Younas et al., 2024; Zhang et al., 2024). Therefore, the phenotypic information on floral organs constitutes a large dataset comprising multidimensional qualitative and quantitative features (De Oliveira, Dias & Dantas, 2011; Khadivi-Khub & Anjam, 2014). Multivariate analysis methods (Khadivi et al., 2025; Sefasi et al., 2025), including correlation analysis, principal component analysis, and cluster analysis, have been widely applied to study the diversity and geographical variation of phenotypic traits, thereby providing a basis for germplasm conservation and breeding decisions (Wang et al., 2023; Chen et al., 2022).

The conservation and management of plant resources are both cumbersome and costly, and establishing a core collection provides a convenient solution for the preservation and effective utilization of plant resources (Sun et al., 2024). The core collection is defined as a subset of germplasm resources that fully represent the phenotypic diversity of the entire population with the least germplasm (Guzmán Luis et al., 2017). It is imperative that the species mass is sufficiently small while maintaining overall phenotypic diversity (Lv et al., 2020). Presently, core collections of various woody plants are constructed based on morphological data (Chen et al., 2025; Shen et al., 2025). Successful examples of core collections for tree species include *Malus pumila* Mill. (Wang et al., 2018), *Prunus sibirica* (Wang et al., 2018), *Robinia pseudoacacia* L. (Guo et al., 2022), and *Camellia sinensis* (L.) Kuntze (Wang et al., 2025). In the genus *Ziziphus*, Chen et al. (2024) developed specialized core collections of sour jujubes focused on fruit and leaf traits for research purposes (Chen et al., 2024). While modern core collections often combine molecular markers with phenotypic data, a phenotype-based approach remains justified. It provides a cost-effective strategy for the initial, large-scale screening of germplasm across diverse sites. More importantly, it directly captures the variation in floral traits of immediate ecological and agronomic relevance. This establishes an essential phenotypic framework, which can then efficiently prioritize accessions for subsequent, targeted molecular analyses in future breeding cycles (Odong et al., 2013; Xu et al., 2016). Thus, constructing a phenotype-based core collection for floral traits represents a critical and practical first step for sour jujube breeding programs.

This study systematically investigated the floral morphology of sour jujube germplasm resources from 30 provenances across eight Chinese provinces. Patterns of phenotypic variation were explored using diversity, correlation, cluster, and principal component analyses. A specialized core collection focused on floral organ research was constructed to facilitate targeted breeding for reproductive traits, support the selection of accessions with optimal floral characteristics for yield and performance. This work aims to offer a scientific basis for the evaluation, breeding, scientific management, and effective utilization of sour jujube germplasm resources.

## Materials and Methods

### Plant materials

**Figure 1:**
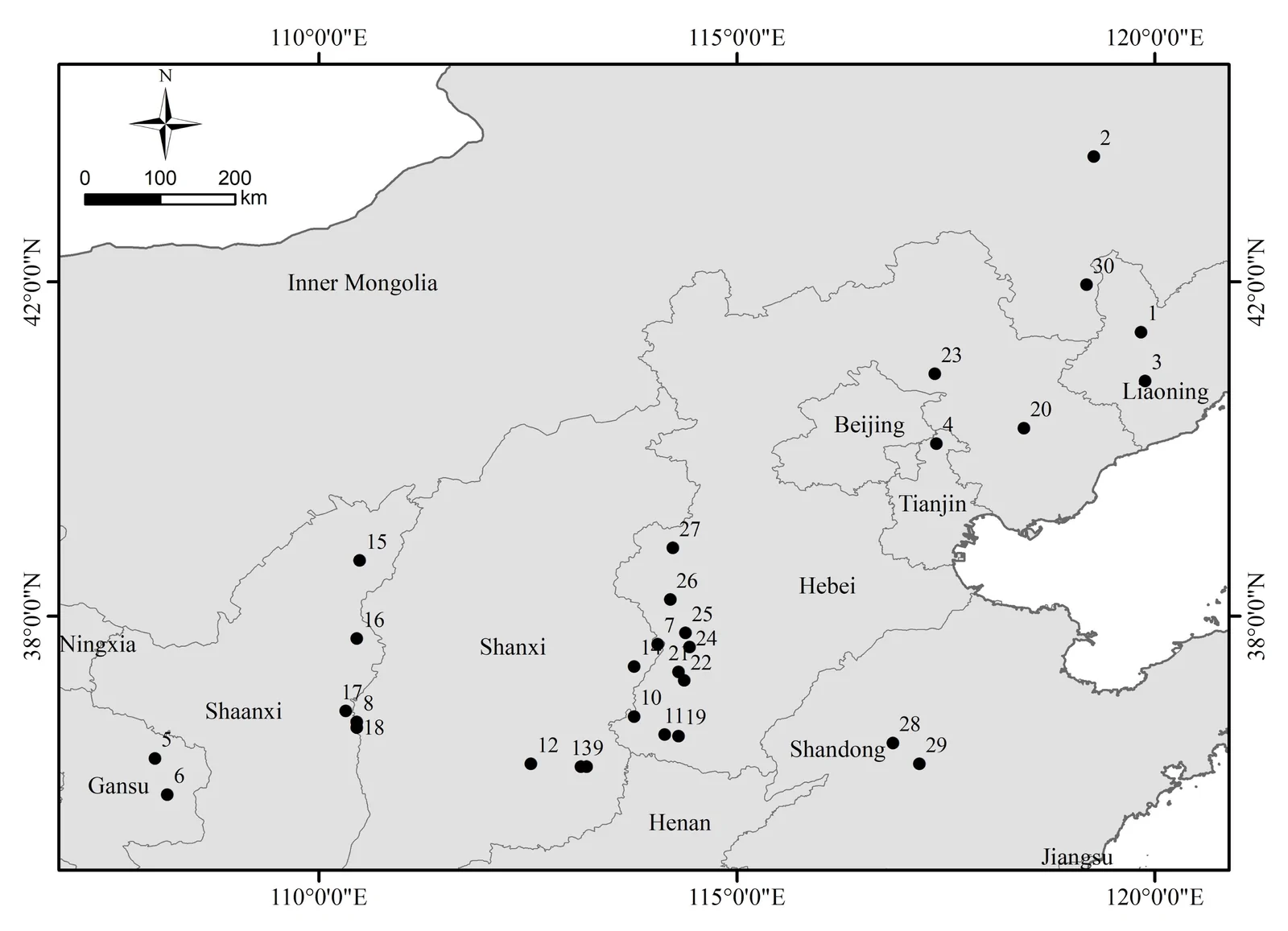
The sampling points distribution map of 30 *Z. jujuba* var. *spinosa* provenances in eight provinces of China. P1: Kazuo, Liaoning (LNKZ); P2: Jianping, Liaoning (LNJP); P3: Jianchang, Liaoning (LNJC); P4: Jizhou, Tianjin (TJJZ); P5: Heshui, Gansu (GSHS); P6: Ningxian, Gansu (GSNX); P7: Jinyang, Shanxi (SXJY); P8: Puxian, Shanxi (SXPX); P9: Qinshui, Shanxi (SXQS); P10: Licheng, Shanxi (SXLC); P11: Pingshun, Shanxi (SXPS); P12: Guxian, Shanxi (SXGX); P13: Gaoping, Shanxi (SXGP); P14: Zuoquan, Shanxi (SXZQ); P15: Jiaxian, Shaanxi (SNJX); P16: Suide, Shaanxi (SNSD); P17: Yanchuan, Shaanxi (SXYC1); P18: Yanchuan, Shaanxi (SNYC2); P19: Fengxing, Hebei (HBFX); P20: Qianxi, Hebei (HBQX); P21: Xindu, Hebei (HBXD); P22: Nieqiu, Hebei (HBNQ); P23: Luanping, Hebei (HBLP); P24: Zanhuang, Hebei (HBZH); P25: Yuanshi, Hebei (HBYS); P26: Luquan, Hebei (HBLQ); P27: Fuping, Hebei (HBFP); P28: Changqing, Shandong (SDCQ); P29: Xintai, Shandong (SDXT); P30: Yuanbaoshan, Neimenggu (MNYB). Map base template (administrative boundary silhouette) source: https://www.naturalearthdata.com/downloads/10m-cultural-vectors/.

The experimental materials comprised 205 clonal sour jujube accessions. In December 2021, scions representing 205 wild sour jujube germplasm accessions were collected from 30 provenances across eight provinces in China (Fig. 1 and Table S1). In May 2022, the collected scions were cleft-grafted onto two-year-old sour jujube rootstocks at the National Forest Germplasm Repository for Sour Jujube of Shenyang Agricultural University (119°49′E, 41°24′N), thereby establishing the corresponding clonal accessions. The rootstocks were planted in north–south rows at a spacing of 2.0 × 3.0 m. The grafted plants grew vigorously and showed no visible signs of pests or diseases. Detailed cultivation conditions are provided in Table S2. The investigation of the organ characteristics was initiated in 2024.

## Experimental Methods

### Observation of phenotypic traits

This study, conducted in 2024, based on the Chinese Jujube Germplasm Resource Descriptors (Ministry of Agriculture of the People’s Republic of China, 2006), investigated a total of seven qualitative traits and 21 quantitative traits. Traits were assigned values according to the phenotypes listed in Table S3. For each clone, we selected three individual trees that were uniform in growth and vigorous for the quantitative trait survey. To ensure phenotypic consistency, all sampled trees were 3-year-old grafted ramets of each clone, cultivated under identical management practices, and were assessed at the full-bloom stage (BBCH 65; about 80% of flowers open) during the 2024 flowering season. Measurements from the 30 flowers per shoot, five shoots per tree, and three trees per clone were first averaged to obtain a single value per tree, then averaged to obtain a clone mean. Clone means were used as the experimental unit for subsequent statistical analyses. For provenance-level analyses, clone means were further averaged to provenance means. The specific measurement methods are as follows.

**Figure 2:**
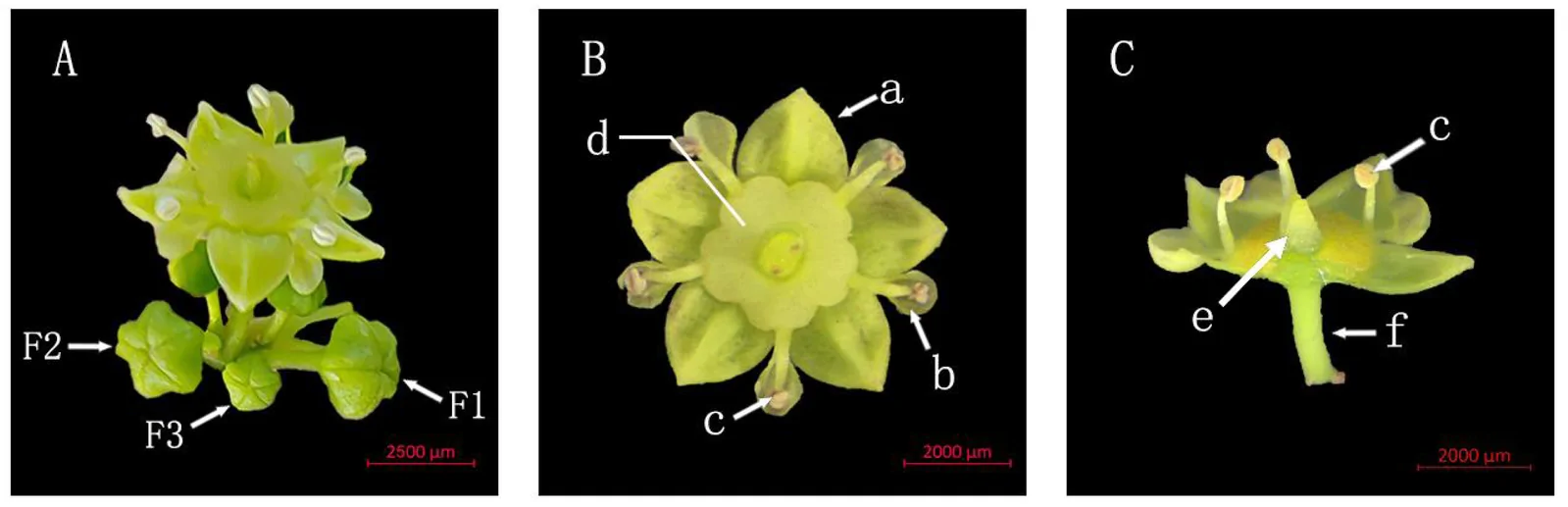
*Ziziphus jujuba* var. *spinosa* flower images. (A) Inflorescence; (B) Front view of the flower; (C) Longitudinal section of the flower; F1, F2, F3 are 1, 2, 3 levels flowers in inflorescence; a: Calyx; b: Petal; c: Stamen; d: Floral disc; e: Pistil; f: Pedicels.

For each sample tree, we selected three bearing shoots from the outer canopy at mid-height (1.5–2.0 m above ground) on the north–south direction of the tree to minimize variation caused by light exposure and microclimate. All selected branches were current-season bearing shoots with uniform diameter (3–5 mm) and length (20–30 cm), and were at the full-bloom stage (BBCH 65). For each branch, we recorded the following floral traits (Fig. 2): number of inflorescences (NOI), number of flowers per inflorescence (NOFI), number of first-level flowers per inflorescence (NOF1), number of second-level flowers per inflorescence (NOF2), number of third-level flowers per inflorescence (NOF3), and flower grade (FG). The flowers located at the fourth to sixth nodes of the selected bearing shoots were measured for pedicels length (PEL) using a digital caliper (±0.01 mm) and the following were observed: flower disc color (FDC), flower disc shape (FDS), petal color (PC), calyx color (CC), petal shape (PS), and calyx shape (CS).

We collected five bearing shoots from each sample tree, placed them in an icebox, and transported them to the laboratory. The flowers were photographed under a stereomicroscope (Stemi 305, Carl Zeiss AG, Jena, Germany) and analyzed using ImageJ software to measure the following quantitative traits: flower diameter (FD), flower disc diameter (FDD), flower area (FA), flower disc area (FDA), flower disc thickness (FDT), stamen length (SL), pistil length (PIL), filament length (FL), petal length (PL), petal width (PW), calyx length (CL), and calyx width (CW). The following ratios were calculated: the calyx aspect ratio (CL/CW), petal aspect ratio (PL/PW), and flower disc proportion (FDA/FA×100%). Thirty flowers were used from each clone.

### Geometeorological data

The climate data used in this study, including annual precipitation (MAP), average temperature in July (T_Jul_), average temperature in January (T_Jan_), and annual mean temperature (MAT), were obtained from the WorldClim database (https://www.worldclim.org/; accessed on 10 November 2024). This database compiles meteorological station records from around the world from 2010 to 2018. It produces global climate raster data at a spatial resolution of 1 km through interpolation. These long-term average climate data represent the stable bioclimatic conditions of each provenance’s native habitat. As all phenotypic evaluations were conducted on grafted clones grown under uniform conditions at a common garden, the observed floral traits reflect phenotypic differentiation among provenances.

Geographic information, including longitude (Long.), latitude (Lat.), and altitude (Alt.), was collected from each original provenance site using handheld GPS devices and high-precision DPA equipment during field surveys in December 2021. These coordinates were then used to extract corresponding climate data from WorldClim raster layers using ArcGIS 10.6 software.

### Data analyses

The base map template we created (administrative boundary outlines) comes from Natural Earth Regional Boundary Silhouettes.

Data were first averaged per tree (30 flowers × 5 shoots) to obtain tree means. For each clone, three tree means were used as replicates in the nested ANOVA. Provenance means were then calculated by averaging clone means within each provenance. Correlation analyses between floral traits and climatic/geographic factors, as well as cluster analysis of 30 provenances, were performed using provenance means. All other analyses were based on clone means.

A nested analysis of variance (nested ANOVA) was used to analyze the phenotypic traits of floral organs in *Ziziphus jujuba* var. *spinosa*. The linear model was: *Y*_*ijk*_ = *μ* + *α*_*i*_ + *β*_*j*(*i*)_ + *ɛ*_*ijk*_, where *Y*_*ijk*_ is the value of the k-th observation for the j-th clone within the i-th provenance, *μ* is the overall mean, *α*_*i*_ is the fixed effect of the i-th provenance, *β*_*j*(*i*)_ is the random effect of the j-th clone within the i-th provenance, and *ɛ*_*ijk*_ is the experimental error of the ijk-th observation. The mean, standard deviation, coefficient of variation (CV), Shannon–Wiener diversity index (H′), and phenotypic differentiation coefficient (V_*st*_) were calculated for each trait.

CV was calculated as:

\begin{eqnarray*}\mathrm{CV}=(\mathrm{SD})/(\mathrm{Mean})\times 100\% \end{eqnarray*}

where SD is the standard deviation of the trait.

H′ was calculated as:

\begin{eqnarray*}{\mathrm{H}}^{{^{\prime}}}=-\sum _{\mathrm{i}=1}^{\mathrm{k}}{\mathrm{P}}_{\mathrm{ i}}\times \ln \nolimits {\mathrm{P}}_{\mathrm{i}} \end{eqnarray*}

where P_i_ represents the frequency of the i-th coded value of a given trait.

V_st_ was calculated as:

\begin{eqnarray*}{\mathrm{V}}_{\mathrm{st}}={\sigma }_{\mathrm{t}/\mathrm{s}}^{2}/({\sigma }_{\mathrm{ t}/\mathrm{s}}^{2}+{\sigma }_{\mathrm{ s}}^{2}) \end{eqnarray*}

where ${\sigma }_{\mathrm{t}/\mathrm{s}}^{2}$ and ${\sigma }_{\mathrm{s}}^{2}$ represent the variance components within and between provenances, respectively.

The CV reflects the degree of dispersion of phenotypic variation for each trait. The H′ was used to assess the diversity of floral morphological traits (Bai et al., 2025). The V_st_ measures the contribution of among-provenance variation to total phenotypic variation (Li et al., 2021). Data processing and basic statistical analyses were performed using Excel 2022 and SPSS 26.0 (IBM Corp., Armonk, NY, USA). Cluster analysis and Pearson correlation analysis were conducted in Origin Pro 2022 to examine phenotypic similarities among sour jujube resources from different provenances and to explore relationships among phenotypic traits as well as between phenotypic traits and environmental factors, with significance thresholds set at *P* < 0.05 and *P* < 0.01. Principal component analysis (PCA) was performed using SPSS 26.0 (Wang et al., 2022).

A specialized core collection for jujube flower organs was constructed using the stepwise clustering method with QGAstation software. Strategies for its development have been explored across various dimensions, including sampling proportion, genetic distance, and sampling methods. Significant differences in phenotypic diversity were detected between the initial and core collections using *F*-tests, *t*-tests, and PCA, to evaluate the effectiveness of the core collection (Chen et al., 2025).

## Results

### Phenotypic trait diversity of sour jujube flowers

#### Diversity analysis of qualitative traits

The seven qualitative traits of sour jujube flowers exhibited 20 distinct phenotypic classes (Table S4). Flower grade (FG)—a composite ordinal trait reflecting inflorescence developmental completeness—showed that Level 2 inflorescences (containing both primary and secondary flowers) were most frequent (47.32%), followed by Level 3 (primary, secondary, and tertiary flowers; 29.76%) and Level 1 (primary flowers only; 22.93%). Biologically, higher flower grade levels represent more complex inflorescence architecture with extended flowering duration and potentially greater reproductive output, as additional flower orders develop sequentially from the primary axis. Petal shape was predominantly broadly obovate (68.29%) *versus* long obovate (31.71%), while calyx shape was mostly obovate-triangular (76.61%). Petal color was dominated by light yellow (69.76%) and yellowish-green (19.02%), and calyx color was overwhelmingly green (88.78%). Flower disc color was most frequently light yellow (46.83%) or apricot yellow (45.85%), and flower disc shape was predominantly circular (47.32%).

The CV for qualitative traits ranged from 17.02% to 48.16% (mean = 37.85%). Six of the seven traits—flower grade, petal shape, petal color, calyx shape, flower disc shape, and flower disc color—exhibited CV >35%, indicating substantial phenotypic variability. Only calyx color was relatively conserved (CV = 17.02%). The H′ ranged from 0.36 to 1.23 (mean = 0.81). Flower grade and flower disc shape showed the highest diversity (H′ > 1.0), reflecting a balanced distribution across phenotypic categories, whereas calyx color exhibited the lowest diversity (H′ = 0.36), consistent with its low CV.

### Diversity analysis of quantitative traits

The 21 quantitative traits showed CV from 7.22% (calyx aspect ratio) to 132.06% (number of third-level flowers per inflorescence), with a mean CV of 25.30% (Table S5). Six traits, including number of flowers per inflorescence, number of first-, second-, and third-level flowers per inflorescence, flower disc thickness, and flower disc area, had CV >25%, indicating high variability in inflorescence architecture and floral disc morphology. In contrast, calyx length, calyx aspect ratio, and petal aspect ratio showed CV <10%, suggesting more conserved traits. The mean H′ for quantitative traits was 1.68, notably higher than that for qualitative traits (0.81), indicating greater evenness and richness in the distribution of quantitative trait phenotypes. The highest H′ values were observed for calyx length (2.10), followed by flower disc diameter, calyx width, and calyx aspect ratio (2.07), whereas the number of first-level flowers per inflorescence showed the lowest diversity (H′ = 0.59).

Nested ANOVA revealed that the variance component among provenances accounted for 65.96% of total phenotypic variation, while within-provenance variance accounted for 25.81% (Table S6). The V_st_ ranged from 46.43% to 84.12% with an average of 71.32%, indicating that 71.32% of the variation in sour jujuba originated from differences among provenances and 28.68% from within provenances. Higher V_st_ values were observed for flower diameter, calyx length, calyx width, and flower disc diameter.

### Correlation analysis

Correlation analysis revealed that geographic factors exerted relatively minor but significant influences on floral morphology. Longitude showed a significant positive correlation with pedicel length (*P* < 0.05), indicating a trend toward elongated pedicels in more eastern provenances. Latitude was significantly positively correlated with flower disc diameter and flower disc area (*P* < 0.05), suggesting that floral discs tend to enlarge with increasing latitude. Altitude demonstrated a significant positive correlation with pistil length (*P* < 0.05).

In contrast, climatic factors exhibited stronger and more extensive associations with floral traits. Mean annual temperature (MAT) showed extremely significant negative correlations with five traits: flower diameter, flower disc diameter, pistil length, flower area, and flower disc area (*P* < 0.01), as well as significant negative correlations with calyx length, calyx width, and petal width (*P* < 0.05). Mean January temperature (T_Jan_) was also highly significantly negatively correlated with flower diameter, flower disc diameter, flower area, and flower disc area (*P* < 0.01), and significantly negatively correlated with calyx width and pistil length (*P* < 0.05). Similarly, mean July temperature (T_Jul_) exhibited extremely significant negative correlations with six traits: flower diameter, flower disc diameter, calyx length, pistil length, flower area, and flower disc area (*P* < 0.01). No significant correlations were detected between annual precipitation (MAP) and any floral trait.

**Figure 3:**
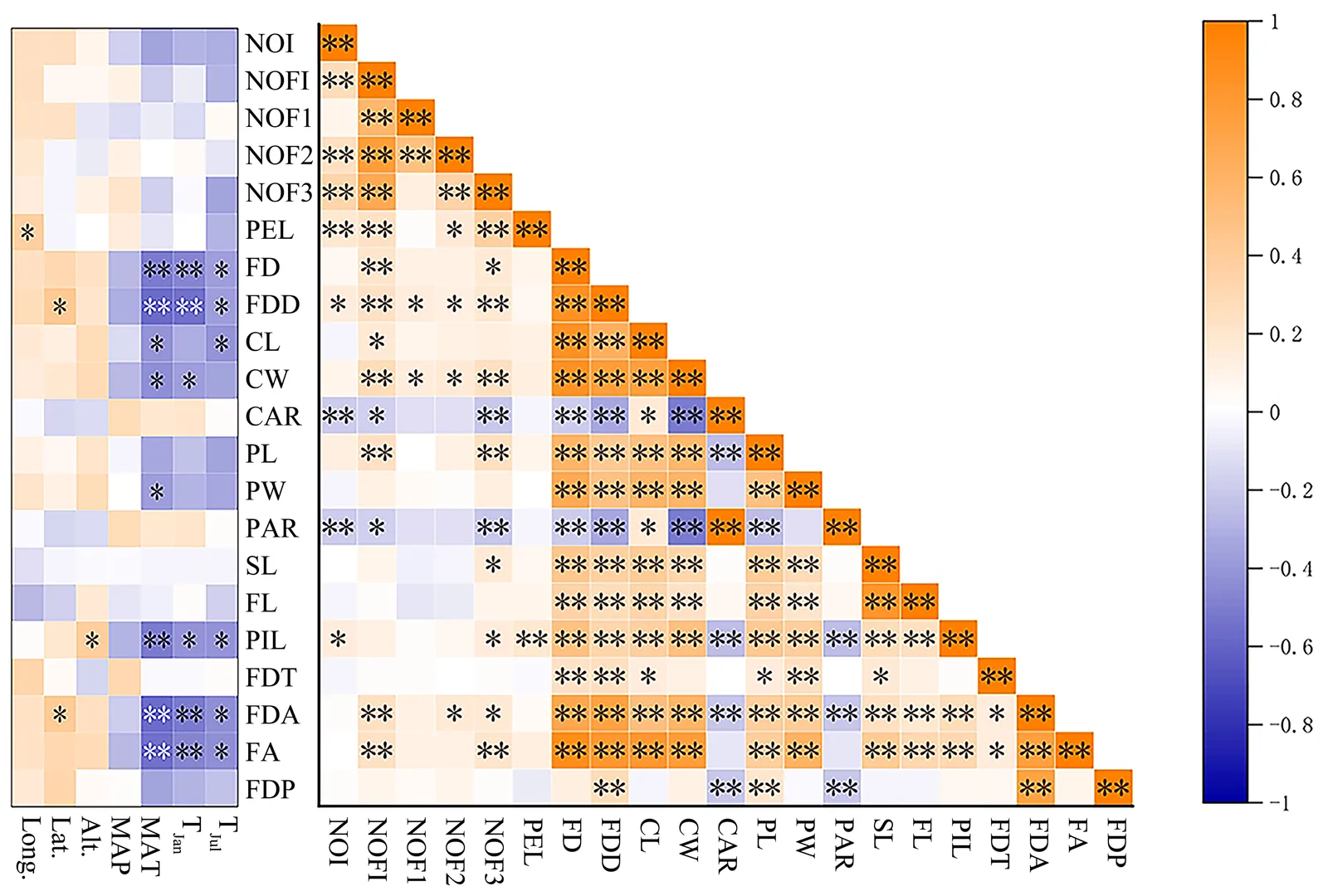
Heatmap of correlation analysis between environmental factors and floral phenotypes in *Z. jujuba* var. *spinosa*. NOI, number of inflorescences; NOFI, number of flowers per inflorescence; NOF1, number of first-level flowers per inflorescence; NOF2, number of second-level flowers per inflorescence; NOF3, number of third-level flowers per inflorescence; PEL, pedicels length; FD, flower diameter; FDD, flower disc diameter; CL, calyx length; CW, calyx width; CAR, calyx aspect ratio; PL, petal length; PW, petal width; PAR, petal aspect ratio; SL, stamen length; FL, filament length; PIL, pistil length; FDT, flower disc thickness; FDA, flower disc area; FA, flower area; FDP, flower disc proportion. MAP, annual precipitation, T_Jul_, average temperature in July; T_Jan_, average temperature in January; MAT, annual mean temperature; Long., longitude; Lat., latitude; Alt., altitude. The * and * * means significant correlation estimates at the level of 0.05 and 0.01, respectively.

Correlation analyses of the quantitative traits of sour jujube flowers from different provenances showed that the size-related traits of wild jujube flowers were strongly correlated with other traits (Fig. 3). Specifically, flower diameter, flower disc diameter, flower area, and flower disc area exhibited highly significant positive correlations (*P* < 0.01) with calyx length, calyx width, petal length, petal width, and number of flowers per inflorescence. Additionally, flower area showed a highly significant negative correlation with petal aspect ratio (*P* < 0.01).

### Cluster analysis

**Figure 4:**
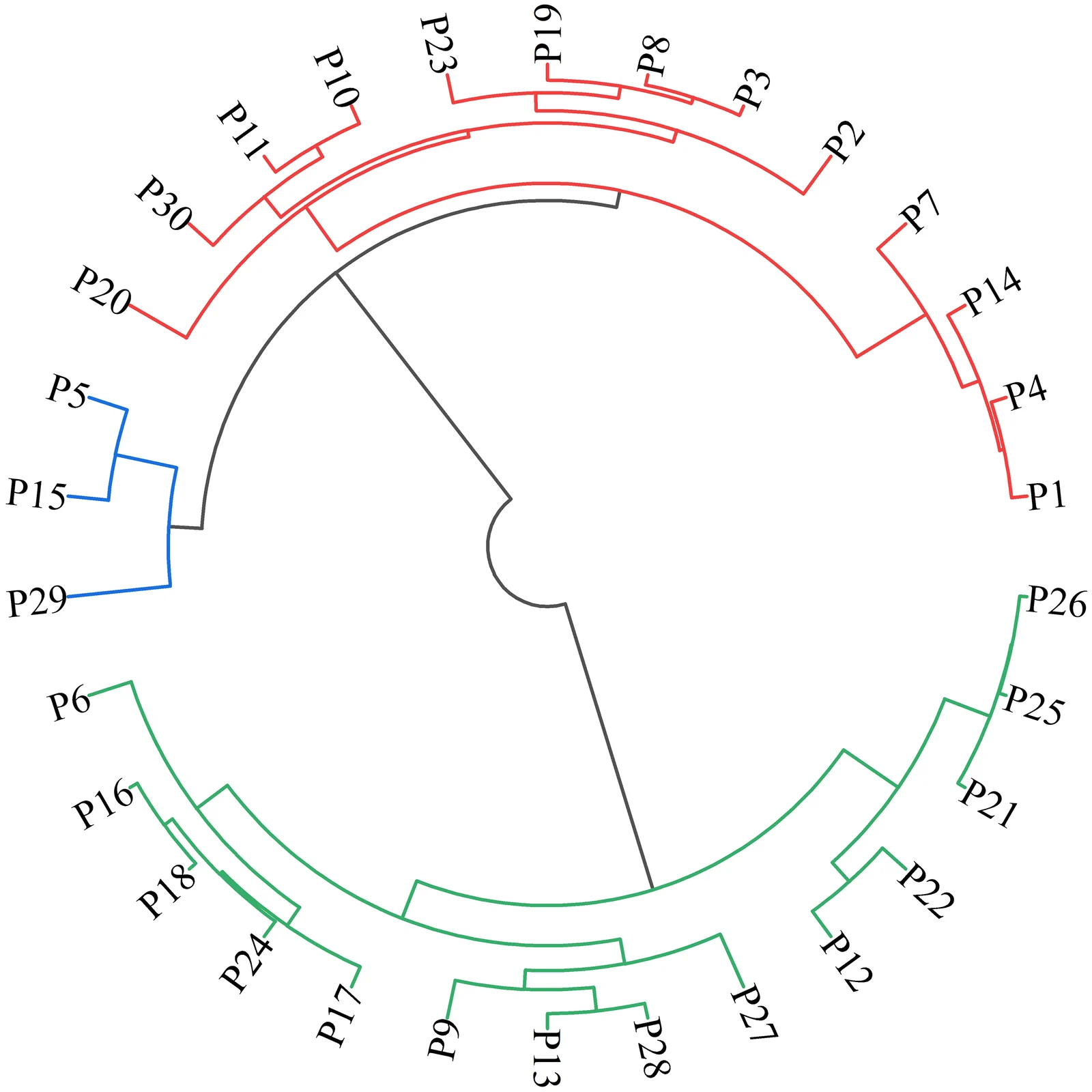
Dendrogram of cluster analysis based on floral phenotypic traits from 30 *Z. jujuba* var. *spinosa* provenances. P1: Kazuo, Liaoning (LNKZ); P2: Jianping, Liaoning (LNJP); P3: Jianchang, Liaoning (LNJC); P4: Jizhou, Tianjin (TJJZ); P5: Heshui, Gansu (GSHS); P6: Ningxian, Gansu (GSNX); P7: Jinyang, Shanxi (SXJY); P8: Puxian, Shanxi (SXPX); P9: Qinshun, Shanxi (SXQS); P10: Licheng, Shanxi (SXLC); P11: Pingshun, Shanxi (SXPS); P12: Guxian, Shanxi (SXGX); P13: Gaoping, Shanxi (SXGP); P14: Zuoquan, Shanxi (SXZQ); P15: Jiaxian, Shaanxi (SNJX); P16: Shunde, Shaanxi (SNSD); P17: Yanchuan, Shaanxi (SXYC1); P18: Yanchuan, Shaanxi (SNYC2); P19: Fengxing, Hebei (HBFX); P20: Qianxi, Hebei (HBQX); P21: Xindu, Hebei (HBXD); P22: Nieqiu, Hebei (HBNQ); P23: Luanping, Hebei (HBLP); P24: Zanhuang, Hebei (HBZH); P25: Yuanshi, Hebei (HBYS); P26: Luquan, Hebei (HBLQ); P27: Fuping, Hebei (HBFP); P28: Changqing, Shandong (SDCQ); P29: Xintai, Shandong (SDXT); P30: Yuanbaoshan, Neimenggu (MNYB).

Cluster analysis divided the 30 germplasm sources into three groups (Fig. 4). Cluster I included Kazuo, Liaoning (LNKZ), Jianchang, Liaoning (LNJC), Jizhou, Tianjin (TJJZ), Jinyang, Shanxi (SXJY), Puxian, Shanxi (SXPX), Licheng, Shanxi (SXLC), Pingshun, Shanxi (SXPS), and Zuoquan, Shanxi (SXZQ). Cluster II includes Heshui, Gansu (GSHS), Jiaxian, Shaanxi (SNJX), and Xintai, Shandong (SDXT). Cluster III includes Jianping, Liaoning (LNJP), Ningxian, Gansu (GSNX), Qinshui, Shanxi (SXQS), Guxian, Shanxi (SNGX), Gaoping, Shanxi (SXGP), Suide, Shaanxi (SNSD), Yanchuan, Shaanxi (SNYC1), Yichuan, Shaanxi (SNYC2), Xindu, Hebei (HBXD), Neiqiu, Hebei (HBNQ), Zanhuang, Hebei (HBZH), Yuanshi, Hebei (HBYS), Luquan, Hebei (HBLQ), and Fuping, Hebei (HEFP). The results of the clustering analysis suggested that the 30 provenances could not be grouped according to geographical distance. The observed discontinuity in variation suggests the potential role of factors such as complex and diverse geographical environments, as well as climatic differences.

**Figure 5:**
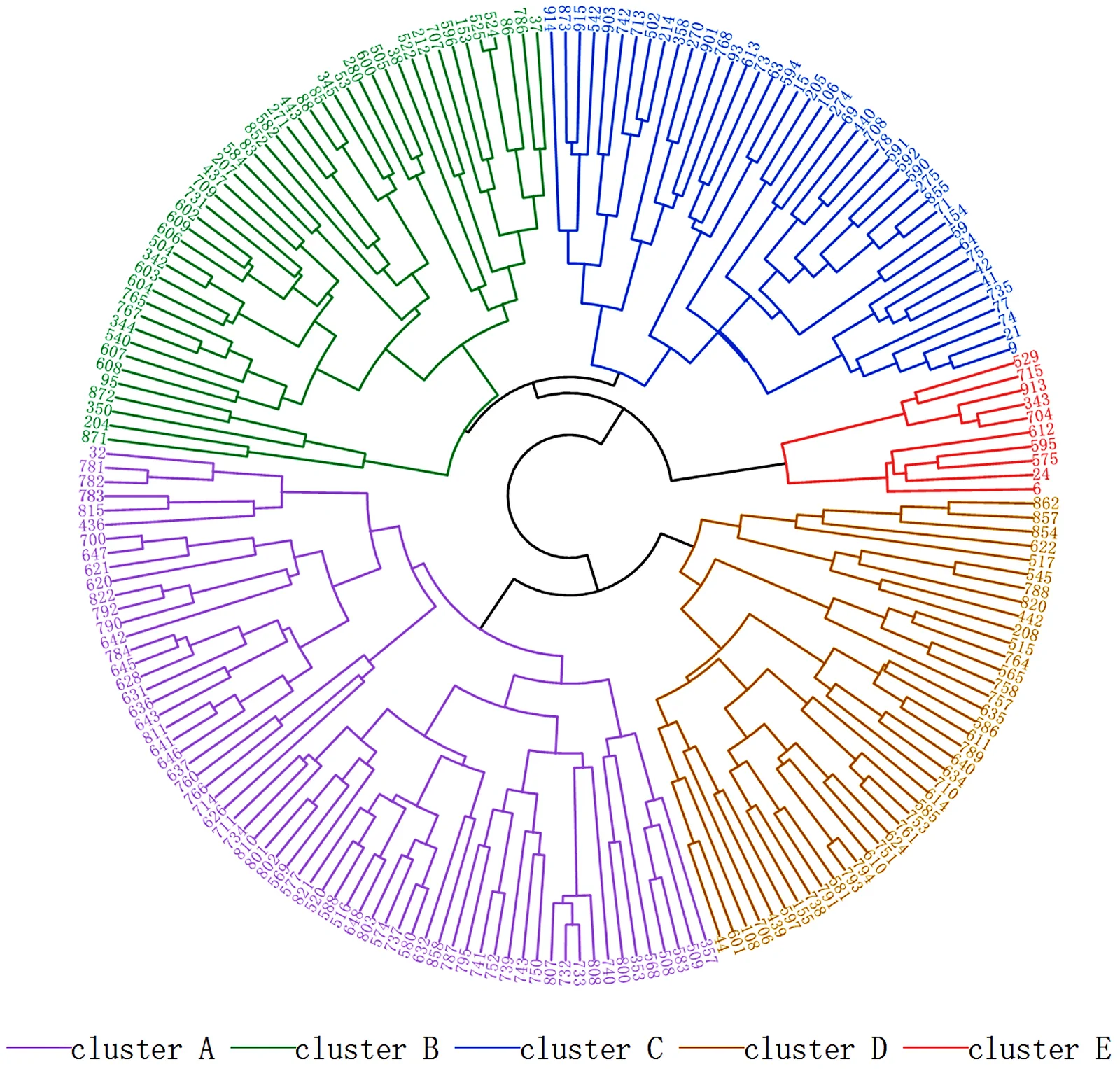
Dendrogram of cluster analysis based on floral phenotypic traits from 205 *Z. jujuba* var. *spinosa* clones.

The 205 clones were subsequently divided into five clusters, and the phenotypic characteristics of each cluster are presented in Table S7 (Fig. 5). Cluster A comprised 65 clones, primarily from HBZH (eight clones), HBYS (six clones), HBXD (seven clones), SNYC1 (seven clones), and SNYC2 (five clones). Overall, this cluster had the smallest characteristics in terms of flower diameter, flower disc diameter, flower stalk length, calyx length, calyx width, flower area, flower disc area, and pistil length. Cluster B included 46 clones, mainly from SXJX (eight clones) and LNKZ (eight clones), with the largest flower diameter, flower disc diameter, calyx length, calyx width, petal length, flower area, flower disc thickness, and pistil length. Cluster C consisted of 43 clones, predominantly LNKZ (17 clones), NMYB (four clones), and SXZQ (four clones). This cluster had the highest number of inflorescences and flowers per inflorescence. Cluster D contained 41 clones, including HBZH (five clones), HBLP (four clones), SXGP (three clones), SXSD(three clones), and SDCP (three clones). The overall characteristics of this cluster were the largest flower stalk length, stamen length, and filament length, but the smallest number of flowers per inflorescence. Cluster E included 10 clones distributed across LNKZ (two clones), HBFX (one clone), SXLC (one clone), and other provenances. This cluster had the smallest mean number of inflorescences, and its flower disc area and proportion were larger than those of the other clusters.

### Principal component analysis

PCA was performed using 21 quantitative descriptive traits as variables (Table S8). Six principal components (PC) with eigenvalues >1 were extracted with contribution rates of 31.67%, 12.98%, 8.99%, 8.37%, 6.28%, and 4.85%. The cumulative contribution rate reached 73.14%, indicating that the six PCs effectively captured essential information of the original traits. In PC 1, the absolute values of the loadings for flower diameter, calyx width, flower area, and flower disc diameter were relatively high, primarily reflecting traits related to flower size. PC 2 was dominated by the absolute loadings of the number of flowers per inflorescence, number of inflorescences, number of first-level flowers per inflorescence, number of second-level flowers per inflorescence, and number of third-level flowers per inflorescence, which mainly represented the traits associated with flower quantity. PC 3 showed high absolute loadings for flower disc area, stamen length, and filament length, primarily reflecting flower disc area- and stamen-related traits. PC 4 was characterized by high absolute loadings for the petal and calyx aspect ratios, mainly representing the shape indices of petals and calyx. PC 5 primarily represented pistil length, whereas PC 6 mainly represented pedicel length.

### Core collection construction and validation

Using a 25% sampling rate, 42 core collection subsets were evaluated. Sampling, genetic distance, and clustering methods were selected based on MD < 20%, CR > 80%, and prioritizing higher CR and VR values. The optimal strategy was “Mahalanobis distance + deviation sampling + shortest distance method” (Table S9). Seven core subsets were generated with this strategy; the 25% sampling subset exhibited the highest VD (71.43%) and CR (98.59%) (Table S10) and was selected as the optimal core subset (D1C2S3-25), comprising 51 accessions.

This 25% proportion was chosen for both statistical and biological reasons. It retained 100% of the phenotypic classes of the seven qualitative traits, captured a greater proportion of peripheral clones (*e.g.*, extreme flower diameter or density), and avoided the unacceptable loss of variation at lower proportions (10–20%) or diminishing returns at higher proportions (30–40%). Thus, 25% represents the minimum sample size that captures the full phenotypic spectrum while eliminating redundancy.

**Figure 6:**
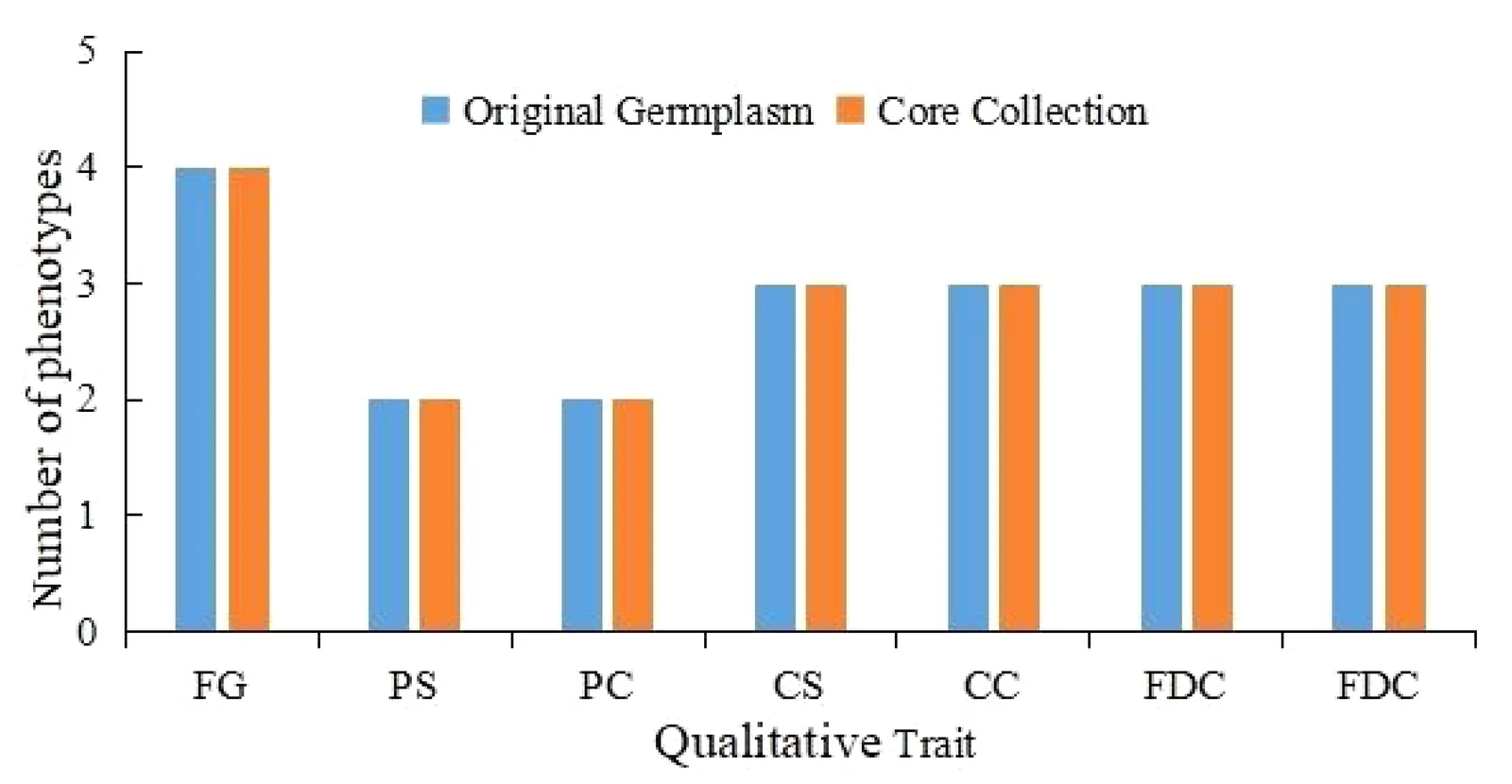
Comparison of phenotypic retention for qualitative traits between core collection and original germplasm. FG, flower grade; PS, petal shape; PC, petal color; CS, calyx shape; CC, calyx color; FDS, flower disc shape; FDC, flower disc color.

**Figure 7:**
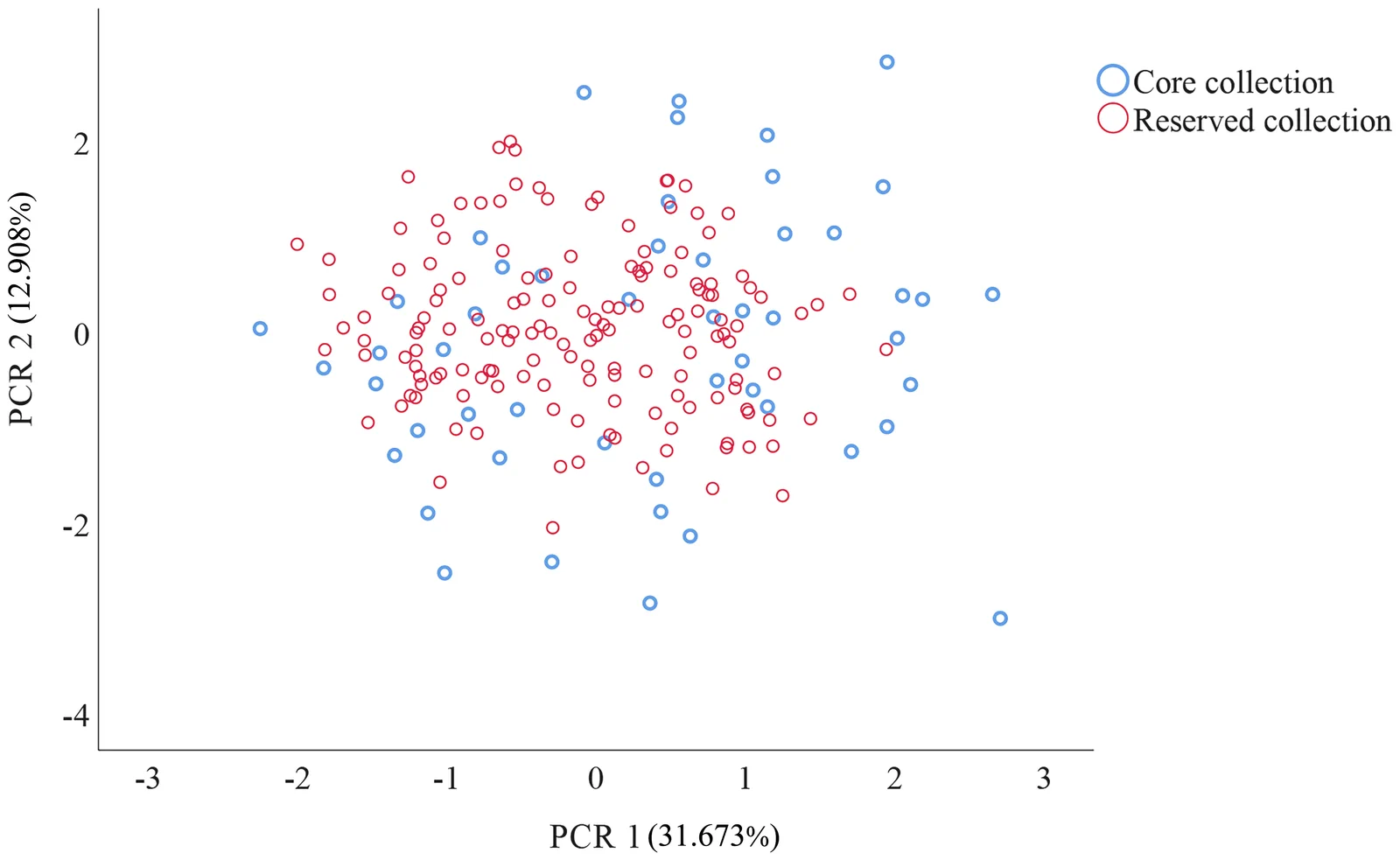
Principal component distribution of core collection and original germplasm.

Validation showed that the core collection retained all seven qualitative trait phenotypes (retention rate 100%, Fig. 6). The *F*-test indicated that, except for six quantitative traits, variances of all other traits were significantly higher in the core collection than in the entire collection (*P* < 0.05). The *t*-test showed no significant differences in trait means except for number of flowers per inflorescence, number of third-level flowers, and flower disc area (Table S11). PCA confirmed that the core collection maintained the distribution of the original germplasm while reducing provenance overlap and included more peripheral clones (Fig. 7), indicating representativeness without redundancy

## Discussion

Research on germplasm resource diversity enables breeders to rapidly access valuable genetic information, with the most straightforward and accessible approach being phenotypic diversity (Moradi et al., 2020). The analysis of sour jujube floral organs revealed rich phenotypic variation. The high mean CV (38.24%) and H′ (0.81) for qualitative traits indicate substantial dispersion and diversity (Pluta, Madry & Sieczko, 2012). Although the mean CV for quantitative traits (25.30%) was lower, suggesting more constrained variation compared to qualitative traits, it still exceeded the variation reported for sour jujube fruits (23.73%; (Qu et al., 2024), highlighting broader diversity in floral structures. Furthermore, a high mean V_st_ (71.32%) was observed, with variation among provenances (65.96%) significantly exceeding that within provenances (25.81%). This value (71.32%) is higher than those reported for other tree species, such as *Juglans mandshurica* Maxim. (50.31%), *Alnus cremastogyne* Burkill (52.61%), and *Tetracentron sinense* Oliv. (56.34%), (Zhang et al., 2021; Zheng et al., 2023; Li et al., 2021), indicating that inter-provenance differences are the primary source of phenotypic diversity in sour jujube. Such strong differentiation may be associated with geographical separation and environmental differences across provenances (Li et al., 2021; Ganopoulos et al., 2016).

Among the various environmental factors, elevation, latitude, and longitude significantly shape phenotypic trait variations in plants (Sun et al., 2021; Yu et al., 2023). For instance, phenotypic variation in the leaves of *Caryopteris mongholica* follows a longitudinal gradient, whereas latitude is the primary factor affecting phenotypic variation in *Quercus mongolica* Fisch. ex Ledeb. and *Quercus variabilis* Blume. In this study, latitude showed a significant positive correlation with flower disc size, while mean annual temperature was negatively correlated with key floral dimensions (flower diameter, flower disc diameter, flower area, and flower disc area). This suggests that sour jujube produces larger flowers under cooler, higher-latitude conditions. This relationship may be associated with several ecological factors, including pollinator behavior and resource allocation under different climatic conditions. In colder environments, where pollinator activity is often limited, larger flowers can enhance visual attraction and reward offer, potentially improving pollination efficiency (Galen, 1999). Additionally, larger floral structures, such as an expanded disc, may aid in capturing solar radiation, slightly warming the floral microenvironment to support pollen tube growth under cooler conditions (Mahmoodi et al., 2021). Finally, this trend could indicate a shift in resource allocation strategy, where plants in shorter growing seasons invest in fewer but larger and potentially more resilient flowers to ensure reproductive success (Alcántara-Ayala et al., 2020).

PCA, which reduces dimensionality by transforming numerous indicators into a few comprehensive indicators (Kombi, Liu & Zhao, 2021), has been widely applied in studies of phenotypic variation in plants such as *Xanthoceras sorbifolium* Bunge (Zhou et al., 2023) and *Schisandra sphenanthera* Rehd. et Wils. (Wang et al., 2022), *Punica granatum* L. (Mishra et al., 2022), and *Carya cathayensis* Sarg. (Wang et al., 2024). For the phenotypic analysis of sour jujube flowers, six principal components were extracted, with a cumulative contribution rate of 73.14%, capturing most of the information from the original floral phenotypic dataset. This high cumulative variance can be attributed to strong multicollinearity and significant correlations among traits (Yang et al., 2022). The absolute loadings of the flower diameter, flower area, and flower disc diameter were relatively high. Combined with the correlation analysis, traits reflecting flower size, such as flower diameter, flower area, and flower disc diameter, showed strong correlations with other traits, including number of flowers per inflorescence, petal width, and calyx length. This further indicates that these traits can serve as key indicators for evaluating the phenotypic characteristics of sour jujube flowers and should be prioritized in future resource assessment efforts. Recent studies in woody perennials have successfully employed such key floral traits as selection indices in breeding programs, demonstrating the translational value of PCA-derived indicators (Wang et al., 2024; Sun et al., 2024).

Cluster analysis can reveal the relationships between varieties with similar characteristics (Safdari & Khadivi, 2021). In this study, 205 sour jujube clones from 30 provenances were classified into five clusters. Cluster A can be utilized for selecting and breeding small-flowered varieties, Cluster B is suitable for breeding large-flowered varieties, Cluster C can serve as parental material for high-yield varieties, Cluster D is characterized by the longest pedicel length and lowest number of flowers per inflorescence, and Cluster E can be used for breeding nectar-rich varieties, demonstrating how phenotypic diversity maps directly onto breeding objectives (Safdari & Khadivi, 2021). Sour jujube flowers are a natural medicinal resource, and their nectar is rare but of high economic value. Breeders can select different clones as parental materials based on practical needs. Notably, the tested sour jujube clones were not strictly clustered according to geographical distance. Although there was some geographical overlap within each cluster, the discontinuity in phenotypic trait variation suggests that factors, such as complex and diverse geographical environments and climatic differences, may have played a role (Ganopoulos et al., 2016). This finding aligns with previous studies on *Elaeagnus angustifolia* L. (Khadivi, 2018; Zhang et al., 2024) and *Persea americana* Mill. (Juma et al., 2021; Kouam, Ngouana & Dountio, 2025), and *Prunus pseudocerasus* Lindl. (Petruccelli et al., 2013). Recent advances in integrating phenotypic and genotypic data have further demonstrated that such discontinuous variation often reflects underlying genomic differentiation rather than simple geographic distance (Wang et al., 2025; Sefasi et al., 2025).

The objective of core collection construction is to maximize the representation of the phenotypic diversity of the original germplasm with a minimal number of accessions, thereby significantly reducing conservation costs and improving the efficiency of germplasm resource preservation (Han et al., 2022; Sun et al., 2024). Constructing a core collection based on phenotypic data is the most straightforward approach and has been widely applied in the development of core collections for tree species such as *Juglans regia* L. (Mahmoodi et al., 2019), *Camellia oleifera* Abel (Zhu et al., 2022), *Robinia pseudoacacia* L. (Guo et al., 2022), and *Prunus dulcis* (Mill.) D. A. Webb (Khojand et al., 2023). We used a stepwise clustering approach to explore the construction of a core collection of sour jujube flowers by considering factors, and the optimal sampling strategy was identified as “25% sampling proportion + Mahalanobis distance + deviation sampling method + nearest neighbor method”, resulting in the selection of 51 core accessions. The optimization of the sampling strategy ensures that this minimal set retains the essential variation—including the ecologically significant correlation between flower size and climate—while maximizing diversity (Deng et al., 2024). This phenotype-based core collection is not an endpoint but a strategic filter. It provides a manageable, highly diverse subset that is ideally suited for subsequent in-depth physiological studies and molecular analyses (Liu et al., 2024). By prioritizing accessions that represent the extreme variation in floral traits, this core collection provides a valuable resource for future studies on the reproductive characteristics and breeding potential of sour jujube.

## Conclusion

This study represents the first comprehensive evaluation of floral organs phenotypic diversity in sour jujube. Our findings reveal a substantial level of phenotypic variation within the germplasm collection, which is predominantly structured by geographical origin. Key floral size traits, such as flower diameter and flower disc diameter, were identified as reliable and practical indicators for germplasm evaluation. Furthermore, the observed geographic patterns in these traits suggest a significant association between environmental factors, particularly temperature, and floral morphology. To capture and conserve this diversity, we successfully constructed a specialized core collection comprising 51 accessions, which effectively represents the overall floral phenotypic variation. This core collection serves as a foundational resource for future studies on floral development and the relationship between floral traits and environmental factors in sour jujube. The identified key phenotypic indicators and the core collection provide practical tools for breeders to select parental lines with desirable floral traits, potentially contributing to improved fruit set and yield. Moreover, our results offer critical insights for the development of targeted conservation strategies, ensuring the preservation of this valuable phenotypic diversity for sustainable utilization.

## Supplemental Information

10.7717/peerj.21708/supp-1 Supplemental Information 1 Phenotypic data of *Ziziphus jujuba* var. *spinosa* flowers LNKZ, Kazuo, Liaoning; LNJP, Jianping, Liaoning; LNJC, Jianchang, Liaoning; TJJZ, Jizhou, Tianjin; GSHS, Heshui, Gansu, GSNX: Ningxian, Gansu; SXJY, Jinyang, Shanxi; SXPX, Puxian, Shanxi; SXQS, Qinshun, Shanxi; SXLC, Licheng, Shanxi; SXPS, Pingshun, Shanxi; SXGX, Guxian, Shanxi; SXGP, Gaoping, Shanxi; SXZQ, Zuoquan, Shanxi; SNJX, Jiaxian, Shaanxi; SNSD, Shunde, Shaanxi; SXYC1, Yanchuan, Shaanxi; SNYC2, Yanchuan, Shaanxi; HBFX, Fengxing, Hebei; HBQX, Qianxi, Hebei; HBXD, Xindu, Hebei; HBNQ, Nieqiu, Hebei; HBLP, Luanping, Hebei; HBZH, Zanhuang, Hebei; HBYS, Yuanshi, Hebei; HBLQ, Luquan, Hebei; HBFP, Fuping, Hebei; SDCQ, Changqing, Shandong; SDXT, Xintai, Shandong; MNYB, Yuanbaoshan, Neimenggu. FG, flower grade; PS, petal shape; PC, petal color; CS, calyx shape; CC, calyx color; FDS, flower disc shape; FDC, flower disc color. NOI, number of inflorescences; NOFI, number of flowers per inflorescence; NOF1, number of first-level flowers per inflorescence; NOF2, number of second-level flowers per inflorescence; NOF3, number of third-level flowers per inflorescence; PEL, pedicels length; FD, flower diameter; FDD, flower disc diameter; CL, calyx length; CW, calyx width; CAR, calyx aspect ratio; PL, petal length; PW, petal width; PAR, petal aspect ratio; SL, stamen length; FL, filament length; PIL, pistil length; FDT, flower disc thickness; FDA, flower disc area; FA, flower area; FDP, flower disc proportion.

10.7717/peerj.21708/supp-2 Supplemental Information 2 The source and sampling of the tested *Ziziphus .jujuba* var. *spinosa* clone P1: Kazuo, Liaoning (LNKZ); P2: Jianping, Liaoning (LNJP); P3: Jianchang, Liaoning (LNJC); P4: Jizhou, Tianjin (TJJZ); P5: Heshui, Gansu (GSHS); P6: Ningxian, Gansu (GSNX); P7: Jinyang, Shanxi (SXJY); P8: Puxian, Shanxi (SXPX); P9: Qinshun, Shanxi (SXQS); P10: Licheng, Shanxi (SXLC); P11: Pingshun, Shanxi (SXPS); P12: Guxian, Shanxi (SXGX); P13: Gaoping, Shanxi (SXGP); P14: Zuoquan, Shanxi (SXZQ); P15: Jiaxian, Shaanxi (SNJX); P16: Shunde, Shaanxi (SNSD); P17: Yanchuan, Shaanxi (SXYC1); P18: Yanchuan, Shaanxi (SNYC2); P19: Fengxing, Hebei (HBFX); P20: Qianxi, Hebei (HBQX); P21: Xindu, Hebei (HBXD); P22: Nieqiu, Hebei (HBNQ); P23: Luanping, Hebei (HBLP); P24: Zanhuang, Hebei (HBZH); P25: Yuanshi, Hebei (HBYS); P26: Luquan, Hebei (HBLQ); P27: Fuping, Hebei (HBFP); P28: Changqing, Shandong (SDCQ); P29: Xintai, Shandong (SDXT); P30: Yuanbaoshan, Neimenggu (MNYB).

10.7717/peerj.21708/supp-3 Supplemental Information 3 Geographical locations and climatic factors of *Z. jujuba* var. *spinosa* provenances LNKZ, Kazuo, Liaoning; LNJP, Jianping, Liaoning; LNJC, Jianchang, Liaoning; TJJZ, Jizhou, Tianjin; GSHS, Heshui, Gansu; GSNX, Ningxian, Gansu; SXJY, Jinyang, Shanxi; SXPX, Puxian, Shanxi; SXQS, Qinshun, Shanxi; SXLC, Licheng, Shanxi; SXPS, Pingshun, Shanxi; SXGX, Guxian, Shanxi; SXGP, Gaoping, Shanxi; SXZQ, Zuoquan, Shanxi; SNJX, Jiaxian, Shaanxi; SNSD, Shunde, Shaanxi; SXYC1, Yanchuan, Shaanxi; SNYC2, Yanchuan, Shaanxi; HBFX, Fengxing, Hebei; HBQX, Qianxi, Hebei; HBXD, Xindu, Hebei; HBNQ, Nieqiu, Hebei; HBLP, Luanping, Hebei; HBZH, Zanhuang, Hebei; HBYS, Yuanshi, Hebei; HBLQ, Luquan, Hebei; HBFP, Fuping, Hebei; SDCQ, Changqing, Shandong; SDXT, Xintai, Shandong; MNYB, Yuanbaoshan, Neimenggu. MAP, annual precipitation, T_Jul_, a verage temperature in July; T_Jan_, a verage temperature in January; MAT, annual mean temperature; Long., longitude; Lat., latitude; Alt., altitude.

10.7717/peerj.21708/supp-4 Supplemental Information 4 Record standard of *Z. jujuba* var. *spinosa* clones in qualitative traits FG, flower grade; PS, petal shape; PC, petal color; CS, calyx shape; CC, calyx color; FDS, flower disc shape; FDC, flower disc color.

10.7717/peerj.21708/supp-5 Supplemental Information 5 Frequency distribution and diversity of qualitative traits in *Z. jujuba* var. *spinosa* flowers FG, flower grade; PS, petal shape; PC, petal color; CS, calyx shape; CC, calyx color; FDS, flower disc shape; FDC, flower disc color.

10.7717/peerj.21708/supp-6 Supplemental Information 6 Diversity of quantitative traits in *Z. jujuba* var. *spinosa* flowers NOI, number of inflorescences; NOFI, number of flowers per inflorescence; NOF1, number of first-level flowers per inflorescence; NOF2, number of second -level flowers per inflorescence; NOF3, number of third -level flowers per inflorescence; PEL, pedicels length; FD, flower diameter; FDD, flower disc diameter; CL, calyx length; CW, calyx width; CAR, calyx aspect ratio; PL, petal length; PW, petal width; PAR, petal aspect ratio; SL, stamen length; FL, filament length; PIL, pistil length; FDT, flower disc thickness; FDA, flower disc area; FA, flower area; FDP, flower disc proportion.

10.7717/peerj.21708/supp-7 Supplemental Information 7 Variance components and phenotypic differentiation coefficients among provenances for floral traits in *Z. jujuba* var. *spinosa* NOI, number of inflorescences; NOFI, number of flowers per inflorescence; NOF1, number of first-level flowers per inflorescence; NOF2, number of second -level flowers per inflorescence; NOF3, number of third -level flowers per inflorescence; PEL, pedicels length; FD, flower diameter; FDD, flower disc diameter; CL, calyx length; CW, calyx width; CAR, calyx aspect ratio; PL, petal length; PW, petal width; PAR, petal aspect ratio; SL, stamen length; FL, filament length; PIL, pistil length; FDT, flower disc thickness; FDA, flower disc area; FA, flower area; FDP, flower disc proportion.

10.7717/peerj.21708/supp-8 Supplemental Information 8 Mean values of floral phenotypic traits among different clusters of *Ziziphus . jujuba* var. *spinosa* clones NOI, number of inflorescences; NOFI, number of flowers per inflorescence; NOF1, number of first-level flowers per inflorescence; NOF2, number of second -level flowers per inflorescence; NOF3, number of third -level flowers per inflorescence; PEL, pedicels length; FD, flower diameter; FDD, flower disc diameter; CL, calyx length; CW, calyx width; CAR, calyx aspect ratio; PL, petal length; PW, petal width; PAR, petal aspect ratio; SL, stamen length; FL, filament length; PIL, pistil length; FDT, flower disc thickness; FDA, flower disc area; FA, flower area; FDP, flower disc proportion.

10.7717/peerj.21708/supp-9 Supplemental Information 9 Principal component analysis of floral phenotypic traits in *Z. jujuba* var. *spinosa*. NOI, number of inflorescences; NOFI: number of flowers per inflorescence; NOF1, number of first-level flowers per inflorescence; NOF2, number of second -level flowers per inflorescence; NOF3, number of third -level flowers per inflorescence; PEL, pedicels length; FD, flower diameter; FDD, flower disc diameter; CL, calyx length; CW, calyx width; CAR, calyx aspect ratio; PL, petal length; PW, petal width; PAR, petal aspect ratio; SL, stamen length; FL, filament length; PIL, pistil length; FDT, flower disc thickness; FDA, flower disc area; FA, flower area; FDP, flower disc proportion.

10.7717/peerj.21708/supp-10 Supplemental Information 10 Trait difference percentages between core collection and original germplasm D1, Euclidean distance; D2, Mahalanobis distance; S1, Random sampling method; S2, Preferred sampling method; S3, Deviation sampling method; C1, Single linkage method; C2, Complete linkage method; C3, Middle distance clustering; C4, Centroid method; C5, Unweighted class average method; C6, Flexible-beta method; C7, Variable method; C8, Ward’s method; MD%, Percentage difference in means; VD%, Percentage difference in variances; CR%, Range compliance rate; VR%, Rate of change of the coefficient of variation.

10.7717/peerj.21708/supp-11 Supplemental Information 11 Trait difference percentages between core collections and original germplasm under seven sampling proportions MD%, Percentage difference in means; VD%, Percentage difference in variances; CR%, Range compliance rate; VR%, Rate of change of the coefficient of variation.

10.7717/peerj.21708/supp-12 Supplemental Information 12 Comparison of genetic diversity between original germplasm and core collection NOI: number of inflorescences; NOFI, number of flowers per inflorescence; NOF1, number of first-level flowers per inflorescence; NOF2, number of second -level flowers per inflorescence; NOF3, number of third -level flowers per inflorescence; PEL, pedicels length; FD, flower diameter; FDD, flower disc diameter; CL, calyx length; CW, calyx width; CAR, calyx aspect ratio; PL, petal length; PW, petal width; PAR, petal aspect ratio; SL,stamen length; FL, filament length; PIL, pistil length; FDT, flower disc thickness; FDA, flower disc area; FA, flower area; FDP, flower disc proportion.

## Additional Information and Declarations

### Data Availability

The following information was supplied regarding data availability: The raw measurements are available in the Supplemental Files.
