# Supplementary material for: Exploring phenotypic diversity and constructing a floral core collection of Ziziphus jujuba var. spinosa
Source: PeerJ. 2026 Sep 24;14:e21708. doi: 10.7717/peerj.21708 (PMC13616194; doi:10.7717/peerj.21708)
Supplement: Supplementary file 1 [file peerj-14-21708-s002.docx]

| No. | Provenance | Sample number | Clone number |
| --- | --- | --- | --- |
| P1 | LNKZ | 30 | 6、9、15、21、24、32、37、38、41、42、44、53、59、63、64、69、71、73、74、75、77、78、83、85、86、88、93、95、106、108 |
| P2 | LNJP | 3 | 153、154、155 |
| P3 | LIJC | 6 | 204、205、207、208、212、214 |
| P4 | TJJZ | 5 | 270、271、274、275、280 |
| P5 | GSHS | 4 | 342、343、344、345 |
| P6 | GSNX | 4 | 350、353、357、358 |
| P7 | SXJY | 6 | 436、437、439、440、442、443 |
| P8 | SXPX | 5 | 502、504、505、508、509 |
| P9 | SXJS | 3 | 515、516、517 |
| P10 | SXLC | 5 | 520、522、524、525、529 |
| P11 | SXPS | 3 | 540、542、545 |
| P12 | SXGX | 5 | 565、568、569、574、575 |
| P13 | SXGP | 8 | 577、580、581、582、583、584、585、586 |
| P14 | SXZQ | 8 | 588、590、591、592、594、595、596、597 |
| P15 | SNJX | 12 | 600、601、602、603、604、606、607、608、609、610、611、612 |
| P16 | SNSD | 7 | 613、614、620、621、622、624、626 |
| P17 | SNYC1 | 8 | 628、631、632、634、635、636、637、640 |
| P18 | SXYC2 | 7 | 641、642、643、645、646、647、648 |
| P19 | GBFX | 8 | 700、704、706、707、708、709、710、711 |
| P20 | HBQX | 3 | 713、714、715 |
| P21 | HBXD | 12 | 731、732、733、734、735、737、738、739、740、741、742、743、 |
| P22 | HBNQ | 4 | 750、751、752、753 |
| P23 | HBLP | 9 | 757、758、760、761、764、765、766、767、768 |
| P24 | HBZH | 14 | 781、782、783、784、786、787、788、789、790、791、792、793、794、795 |
| P25 | HBYS | 6 | 800、801、802、803、807、808 |
| P26 | HBLQ | 3 | 810、811、815 |
| P27 | HBFP | 3 | 820、821、822 |
| P28 | SDCQ | 6 | 852、854、855、857、858、862 |
| P29 | SDXT | 3 | 871、872、873 |
| P30 | NMYB | 5 | 901、903、913、914、915 |
