# Supplementary material for: Exploring phenotypic diversity and constructing a floral core collection of Ziziphus jujuba var. spinosa
Source: PeerJ. 2026 Sep 24;14:e21708. doi: 10.7717/peerj.21708 (PMC13616194; doi:10.7717/peerj.21708)
Supplement: Supplementary file 2 [file peerj-14-21708-s003.docx]

| Provenance | Long. (E) | Lat. (N) | Alt. (m) | MAP (mm) | MAT (℃) | T_Jan_ (℃) | T_Jul_ (℃) |
| --- | --- | --- | --- | --- | --- | --- | --- |
| LNKZ | 119°49′ | 41°24′ | 407.30 | 543.79 | 8.60 | -7.27 | 24.15 |
| LNJP | 119°16′ | 43°30′ | 512.33 | 416.23 | 6.87 | -10.02 | 23.80 |
| LIJC | 119°52′ | 40°49′ | 443.00 | 543.79 | 8.70 | -6.47 | 23.83 |
| TJJZ | 117°22′ | 40°04′ | 149.75 | 540.56 | 11.25 | -3.87 | 25.70 |
| GSHS | 108°01′ | 36°18′ | 1160.25 | 610.08 | 10.09 | -2.51 | 22.54 |
| GSNX | 108°10′ | 35°52′ | 1146.75 | 610.08 | 10.18 | -2.29 | 22.44 |
| SXJY | 114°10′ | 37°40′ | 981.67 | 707.73 | 8.44 | -4.17 | 20.74 |
| SXPX | 110°27′ | 36°43′ | 1092.80 | 566.57 | 11.32 | -2.90 | 24.92 |
| SXJS | 113°12′ | 36°12′ | 907.33 | 598.18 | 10.29 | -2.48 | 22.02 |
| SXLC | 113°46′ | 36°48′ | 925.00 | 679.10 | 10.29 | -2.67 | 22.52 |
| SXPS | 114°07′ | 36°03′ | 480.00 | 611.06 | 11.24 | -1.85 | 23.48 |
| SXGX | 112°31′ | 36°13′ | 888.20 | 673.40 | 11.29 | -1.86 | 23.65 |
| SXGP | 113°10′ | 36°12′ | 912.75 | 598.18 | 10.29 | -2.48 | 22.02 |
| SXZQ | 113°46′ | 37°24′ | 900.00 | 672.07 | 9.24 | -3.49 | 21.40 |
| SNJX | 110°28′ | 38°40′ | 777.55 | 541.25 | 9.61 | -5.38 | 24.48 |
| SNSD | 110°27′ | 37°43′ | 1041.00 | 535.82 | 10.41 | -4.18 | 24.78 |
| SNYC1 | 110°19′ | 36°52′ | 631.33 | 586.98 | 11.70 | -2.66 | 25.46 |
| SXYC2 | 110°27′ | 36°34′ | 466.20 | 566.57 | 11.38 | -2.82 | 24.87 |
| GBFX | 114°24′ | 36°34′ | 177.38 | 611.06 | 14.03 | -0.28 | 26.88 |
| HBQX | 118°25′ | 40°15′ | 145.00 | 696.80 | 10.16 | -4.56 | 24.84 |
| HBXD | 114°24′ | 37°13′ | 198.94 | 707.73 | 12.76 | -1.12 | 25.71 |
| HBNQ | 114°22′ | 37°23′ | 167.99 | 707.73 | 13.33 | -0.84 | 26.43 |
| HBLP | 117°22′ | 40°54′ | 518.17 | 530.19 | 8.11 | -7.12 | 22.87 |
| HBZH | 114°28′ | 37°36′ | 110.10 | 707.73 | 13.55 | -0.98 | 26.82 |
| HBYS | 114°25′ | 37°46′ | 137.18 | 707.73 | 13.24 | -1.15 | 26.45 |
| HBLQ | 114°12′ | 38°12′ | 232.40 | 573.15 | 12.66 | -1.64 | 25.98 |
| HBFP | 114°13′ | 38°49′ | 258.29 | 650.76 | 11.08 | -2.65 | 24.32 |
| SDCQ | 116°25′ | 36°25′ | 117.64 | 799.48 | 13.65 | -0.33 | 26.53 |
| SDXT | 117°10′ | 36°13′ | 217.67 | 799.48 | 12.60 | -0.82 | 25.25 |
| NMYB | 119°10′ | 41°58′ | 599.83 | 518.18 | 7.51 | -8.76 | 23.71 |
