# Supplementary material for: Exploring phenotypic diversity and constructing a floral core collection of Ziziphus jujuba var. spinosa
Source: PeerJ. 2026 Sep 24;14:e21708. doi: 10.7717/peerj.21708 (PMC13616194; doi:10.7717/peerj.21708)
Supplement: Supplementary file 3 [file peerj-14-21708-s004.docx]

| No. | Quality traits | Criterion for recording |
| --- | --- | --- |
| 1 | FG | （1）level 1（2）level 2（3）level 3（4）level 4 |
| 2 | PS | （1）Broadly obovate（2）Long obovate |
| 3 | PC | （1）**Light yellow**（2）**Yellowish-green**（3）**Green** |
| 4 | CS | （1）**Broadly obovate-triangular**（2）**Long obovate-triangular**（3）**Subtriangular** |
| 5 | CC | （1）Light yellow（2）**Yellowish-green** |
| 6 | FDS | （1）**Orbicular**（2）**Angular**（3）**Pentagonal** |
| 7 | FDC | （1）**Light yellow**（2）**Apricot yellow**（3）**Orange-yellow** |
