# Supplementary material for: Exploring phenotypic diversity and constructing a floral core collection of Ziziphus jujuba var. spinosa
Source: PeerJ. 2026 Sep 24;14:e21708. doi: 10.7717/peerj.21708 (PMC13616194; doi:10.7717/peerj.21708)
Supplement: Supplementary file 4 [file peerj-14-21708-s005.docx]

| Number | Quality trait | Frequency distribution % | | | | CV | *H'* |
| --- | --- | --- | --- | --- | --- | --- | --- |
|  |  | 1 | 2 | 3 | 4 |  |  |
| 1 | FG | 12.68 | 47.32 | 29.76 | 10.24 | 35.46 | 1.23 |
| 2 | PS | 68.29 | 31.71 |  |  | 35.40 | 0.62 |
| 3 | PC | 69.76 | 19.02 | 11.22 |  | 48.16 | 0.81 |
| 4 | CS | 76.61 | 15.12 | 9.27 |  | 47.00 | 0.71 |
| 5 | CC | 11.22 | 88.78 |  |  | 17.02 | 0.36 |
| 6 | FDS | 47.32 | 30.73 | 21.95 |  | 43.13 | 1.05 |
| 7 | FDC | 46.83 | 45.85 | 7.32 |  | 38.77 | 0.91 |
| Mean |  |  |  |  |  | 37.85 | 0.81 |
