# Supplementary material for: Exploring phenotypic diversity and constructing a floral core collection of Ziziphus jujuba var. spinosa
Source: PeerJ. 2026 Sep 24;14:e21708. doi: 10.7717/peerj.21708 (PMC13616194; doi:10.7717/peerj.21708)
Supplement: Supplementary file 5 [file peerj-14-21708-s006.docx]

| Traits | Mean | Max | Min. | Range | SD | CV (%) | H′ |
| --- | --- | --- | --- | --- | --- | --- | --- |
| NOI | 10.61 | 16.00 | 7.00 | 9.00 | 1.49 | 14.00 | 1.66 |
| NOFI | 3.68 | 11.00 | 1.00 | 10.00 | 1.33 | 36.19 | 1.52 |
| NOF1 | 1.24 | 2.00 | 0.00 | 2.00 | 0.44 | 35.51 | 0.59 |
| NOF2 | 1.02 | 4.00 | 0.00 | 4.00 | 0.58 | 56.72 | 0.83 |
| NOF3 | 0.42 | 2.00 | 0.00 | 2.00 | 0.56 | 132.06 | 0.78 |
| PEL | 1.99 | 6.13 | 4.15 | 3.14 | 0.73 | 23.19 | 1.86 |
| FD | 5.82 | 7.14 | 4.65 | 2.49 | 0.57 | 10.74 | 2.04 |
| FDD | 2.45 | 3.39 | 1.74 | 1.65 | 0.28 | 11.59 | 2.07 |
| CL | 1.80 | 2.24 | 1.42 | 0.82 | 0.17 | 9.49 | 2.10 |
| CW | 1.65 | 2.08 | 1.30 | 0.78 | 0.18 | 10.92 | 2.07 |
| CAR | 1.09 | 1.28 | 0.94 | 0.35 | 0.08 | 7.22 | 2.07 |
| PL | 1.57 | 3.45 | 1.09 | 2.36 | 0.25 | 15.75 | 1.85 |
| PW | 0.91 | 1.57 | 0.53 | 1.04 | 0.15 | 17.06 | 1.98 |
| PAR | 1.76 | 3.87 | 0.94 | 2.93 | 0.29 | 7.22 | 1.91 |
| SL | 1.62 | 2.10 | 1.06 | 1.04 | 0.18 | 11.27 | 2.03 |
| FL | 1.08 | 1.55 | 0.64 | 0.91 | 0.16 | 14.49 | 2.01 |
| PIL | 0.80 | 1.20 | 0.19 | 1.01 | 0.16 | 20.39 | 2.05 |
| FDT | 0.26 | 1.30 | 0.12 | 1.18 | 0.10 | 38.04 | 1.49 |
| FDA | 4.81 | 8.95 | 2.35 | 6.59 | 1.20 | 25.05 | 1.98 |
| FA | 19.32 | 31.19 | 12.83 | 18.35 | 3.37 | 17.42 | 2.01 |
| FDP | 0.25 | 0.51 | 0.16 | 0.35 | 0.04 | 17.08 | 2.05 |
| Mean |  |  |  |  |  | 25.30 | 1.68 |
