# Supplementary material for: Exploring phenotypic diversity and constructing a floral core collection of Ziziphus jujuba var. spinosa
Source: PeerJ. 2026 Sep 24;14:e21708. doi: 10.7717/peerj.21708 (PMC13616194; doi:10.7717/peerj.21708)
Supplement: Supplementary file 6 [file peerj-14-21708-s007.docx]

| Traits | Variance component | | | Percentage of Variance component (%) | | | V_st_/% |
| --- | --- | --- | --- | --- | --- | --- | --- |
|  | Within provenances | Among provenances | Random error | Within provenances | Among provenances | Random  error |  |
| NOI | 4.495 | 9.389 | 1.218 | 29.76 | 62.17 | 8.07 | 67.62 |
| NOFI | 3.581 | 9.804 | 0.481 | 25.83 | 70.71 | 3.47 | 73.25 |
| NOF1 | 0.362 | 1.278 | 0.075 | 21.11 | 74.52 | 4.37 | 77.93 |
| NOF2 | 0.729 | 1.865 | 0.112 | 26.94 | 68.92 | 4.14 | 71.90 |
| NOF3 | 0.764 | 1.359 | 0.129 | 33.93 | 60.35 | 5.73 | 64.01 |
| PEL | 1.206 | 4.054 | 0.070 | 22.63 | 76.06 | 1.31 | 77.07 |
| FD | 0.669 | 3.545 | 0.081 | 15.58 | 82.54 | 1.89 | 84.12 |
| FDD | 0.181 | 0.700 | 0.026 | 19.96 | 77.18 | 2.87 | 79.46 |
| CL | 0.064 | 0.277 | 0.017 | 17.88 | 77.37 | 4.75 | 81.23 |
| CW | 0.072 | 0.307 | 0.021 | 18.00 | 76.75 | 5.25 | 81.00 |
| CAR | 0.028 | 0.036 | 0.016 | 35.00 | 45.00 | 20.00 | 56.25 |
| PL | 0.140 | 0.318 | 0.069 | 26.57 | 60.34 | 13.09 | 69.43 |
| PW | 0.072 | 0.215 | 0.039 | 22.09 | 65.95 | 11.96 | 74.91 |
| PAR | 0.188 | 0.566 | 0.075 | 22.68 | 68.28 | 9.05 | 75.07 |
| SL | 0.096 | 0.181 | 0.023 | 32.00 | 60.33 | 7.67 | 65.34 |
| FL | 0.071 | 0.124 | 0.020 | 33.02 | 57.67 | 9.30 | 63.59 |
| PIL | 0.057 | 0.183 | 0.008 | 22.98 | 73.79 | 3.23 | 76.25 |
| FDT | 0.031 | 0.063 | 0.004 | 31.63 | 64.29 | 4.08 | 67.02 |
| FDA | 6.306 | 14.250 | 4.820 | 24.85 | 56.16 | 18.99 | 69.32 |
| FA | 30.549 | 99.587 | 2.563 | 23.02 | 75.05 | 1.93 | 76.53 |
| FDP | 0.015 | 0.013 | 0.013 | 36.59 | 31.71 | 31.71 | 46.43 |
| Mean | 2.370 | 7.050 | 0.470 | 25.81 | 65.96 | 8.23 | 71.32 |
