# Supplementary material for: Exploring phenotypic diversity and constructing a floral core collection of Ziziphus jujuba var. spinosa
Source: PeerJ. 2026 Sep 24;14:e21708. doi: 10.7717/peerj.21708 (PMC13616194; doi:10.7717/peerj.21708)
Supplement: Supplementary file 7 [file peerj-14-21708-s008.docx]

| Traits | **Clusters** | | | | |
| --- | --- | --- | --- | --- | --- |
|  | A | B | C | D | E |
| NOI | 10.89 | 10.28 | 11.30 | 10.10 | 9.50 |
| NOFI | 3.69 | 3.39 | 4.65 | 3.10 | 3.20 |
| NOF1 | 1.25 | 1.02 | 1.67 | 1.07 | 1.10 |
| NOF2 | 1.08 | 0.85 | 1.47 | 0.66 | 1.10 |
| NOF3 | 0.38 | 0.46 | 0.56 | 0.39 | 0.10 |
| PEL | 5.25 | 6.33 | 6.09 | 5.87 | 5.75 |
| FD | 2.98 | 3.07 | 3.24 | 3.37 | 3.12 |
| FDD | 2.22 | 2.73 | 2.58 | 2.40 | 2.41 |
| CL | 1.63 | 1.90 | 1.87 | 1.85 | 1.83 |
| CW | 1.49 | 1.83 | 1.77 | 1.60 | 1.62 |
| CAR | 1.10 | 1.04 | 1.06 | 1.16 | 1.14 |
| PL | 1.47 | 1.71 | 1.59 | 1.58 | 1.47 |
| PW | 0.79 | 1.02 | 0.97 | 0.91 | 0.88 |
| PAR | 1.89 | 1.70 | 1.65 | 1.76 | 1.67 |
| SL | 1.56 | 1.71 | 1.52 | 1.72 | 1.53 |
| FL | 1.04 | 1.15 | 0.99 | 1.18 | 1.00 |
| PIL | 3.90 | 5.66 | 5.12 | 4.48 | 6.72 |
| FDT | 16.08 | 22.29 | 20.62 | 19.65 | 19.70 |
| FDA | 0.24 | 0.31 | 0.27 | 0.25 | 0.24 |
| FA | 0.71 | 0.86 | 0.85 | 0.81 | 0.82 |
| FDP | 0.24 | 0.25 | 0.25 | 0.23 | 0.35 |
