# Supplementary material for: Exploring phenotypic diversity and constructing a floral core collection of Ziziphus jujuba var. spinosa
Source: PeerJ. 2026 Sep 24;14:e21708. doi: 10.7717/peerj.21708 (PMC13616194; doi:10.7717/peerj.21708)
Supplement: Supplementary file 8 [file peerj-14-21708-s009.docx]

| Traits | Principal component | | | | | |
| --- | --- | --- | --- | --- | --- | --- |
|  | PC1 | PC2 | PC3 | PC4 | PC5 | PC6 |
| FD | 0.941 | -0.176 | -0.075 | 0.001 | -0.040 | -0.026 |
| CW | 0.886 | -0.003 | -0.076 | -0.104 | -0.259 | -0.154 |
| FA | 0.875 | -0.178 | -0.194 | 0.064 | 0.057 | 0.142 |
| FDD | 0.855 | -0.048 | -0.121 | -0.182 | 0.040 | 0.053 |
| CL | 0.805 | -0.249 | -0.088 | 0.247 | 0.063 | 0.031 |
| FDA | 0.754 | 0.005 | -0.190 | -0.451 | 0.284 | 0.273 |
| PW | 0.691 | -0.281 | -0.298 | 0.199 | -0.207 | -0.076 |
| PL | 0.660 | 0.050 | 0.425 | -0.176 | -0.083 | -0.218 |
| PIL | 0.532 | -0.013 | 0.176 | -0.015 | -0.405 | -0.116 |
| NOFI | 0.382 | 0.790 | -0.064 | 0.267 | 0.202 | -0.080 |
| NOF2 | 0.249 | 0.730 | -0.187 | 0.219 | 0.221 | -0.125 |
| NOF3 | 0.350 | 0.593 | 0.201 | 0.238 | -0.109 | 0.265 |
| NOF1 | 0.188 | 0.529 | -0.350 | 0.186 | 0.278 | -0.392 |
| NOI | 0.139 | 0.478 | 0.189 | 0.003 | -0.290 | 0.211 |
| PAR | -0.089 | 0.328 | 0.705 | -0.392 | 0.138 | -0.148 |
| FL | 0.423 | -0.282 | 0.612 | 0.255 | 0.293 | -0.073 |
| SL | 0.527 | -0.250 | 0.561 | 0.259 | 0.279 | -0.077 |
| FDP | 0.227 | 0.185 | -0.073 | -0.750 | 0.377 | 0.264 |
| CAR | -0.279 | -0.325 | -0.013 | 0.478 | 0.481 | 0.293 |
| FDT | 0.244 | -0.139 | -0.079 | 0.064 | 0.252 | 0.016 |
| PEL | 0.182 | 0.302 | 0.209 | 0.288 | -0.254 | 0.591 |
| Eigenvalue | 6.651 | 2.726 | 1.887 | 1.757 | 1.320 | 1.018 |
| Contributive percentage (%) | 31.673 | 12.980 | 8.986 | 8.369 | 6.284 | 4.847 |
| Total percentage (%) | 31.673 | 44.653 | 53.639 | 62.008 | 68.292 | 73.139 |
