# Supplementary material for: Exploring phenotypic diversity and constructing a floral core collection of Ziziphus jujuba var. spinosa
Source: PeerJ. 2026 Sep 24;14:e21708. doi: 10.7717/peerj.21708 (PMC13616194; doi:10.7717/peerj.21708)
Supplement: Supplementary file 9 [file peerj-14-21708-s010.docx]

| Core collection | MD% | VD% | CR% | VR% |
| --- | --- | --- | --- | --- |
| D1S1C1 | 4.76 | 47.62 | 97.27 | 117.65 |
| D1S1C2 | 0.00 | 23.81 | 89.52 | 110.86 |
| D1S1C3 | 0.00 | 9.52 | 91.86 | 109.63 |
| D1S1C4 | 0.00 | 14.29 | 86.37 | 108.08 |
| D1S1C5 | 0.00 | 4.76 | 81.96 | 106.62 |
| D1S1C6 | 0.00 | 14.29 | 82.48 | 103.91 |
| D1S1C7 | 0.00 | 14.29 | 85.07 | 109.71 |
| D1S1C8 | 0.00 | 52.38 | 100.00 | 121.97 |
| D1S2C1 | 0.00 | 61.90 | 100.00 | 121.81 |
| D1S2C2 | 0.00 | 52.38 | 100.00 | 121.95 |
| D1S2C3 | 0.00 | 42.86 | 100.00 | 119.45 |
| D1S2C4 | 4.76 | 57.14 | 100.00 | 120.30 |
| D1S2C5 | 0.00 | 52.38 | 100.00 | 122.77 |
| D1S2C6 | 0.00 | 52.38 | 100.00 | 121.06 |
| D1S2C7 | 0.00 | 57.14 | 100.00 | 121.83 |
| D1S2C8 | 0.00 | 52.38 | 100.00 | 121.97 |
| D1S3C1 | 0.00 | 66.67 | 95.21 | 123.97 |
| D1S3C2 | 0.00 | 57.14 | 92.56 | 119.86 |
| D1S3C3 | 0.00 | 23.81 | 94.48 | 117.76 |
| D1S3C4 | 0.00 | 52.38 | 94.82 | 120.49 |
| D1S3C5 | 0.00 | 38.10 | 92.62 | 120.20 |
| D1S3C6 | 0.00 | 23.81 | 90.85 | 120.37 |
| D1S3C7 | 0.00 | 23.81 | 90.85 | 120.37 |
| D1S3C8 | 0.00 | 38.10 | 93.30 | 121.49 |
| D2S1C1 | 9.52 | 38.10 | 96.51 | 116.28 |
| D2S1C2 | 0.00 | 9.52 | 87.40 | 111.12 |
| D2S1C3 | 4.76 | 4.76 | 89.15 | 107.12 |
| D2S1C4 | 4.76 | 57.14 | 96.02 | 118.68 |
| D2S1C5 | 0.00 | 14.29 | 91.51 | 114.10 |
| D2S1C6 | 0.00 | 14.29 | 87.30 | 107.66 |
| D2S1C7 | 0.00 | 4.76 | 83.23 | 104.16 |
| D2S1C8 | 0.00 | 28.57 | 89.32 | 109.16 |
| D2S2C1 | 0.00 | 71.43 | 100.00 | 122.13 |
| D2S2C2 | 0.00 | 47.62 | 100.00 | 122.07 |
| D2S2C3 | 0.00 | 47.62 | 100.00 | 121.50 |
| D2S2C4 | 0.00 | 66.67 | 100.00 | 121.00 |
| D2S2C5 | 0.00 | 52.38 | 100.00 | 121.68 |
| D2S2C6 | 0.00 | 42.86 | 100.00 | 119.89 |
| D2S2C7 | 0.00 | 38.10 | 100.00 | 120.62 |
| D2S2C8 | 0.00 | 42.86 | 100.00 | 120.73 |
| D2S3C1 | 9.52 | 71.43 | 98.59 | 123.84 |
| D2S3C2 | 0.00 | 66.67 | 93.95 | 123.08 |
| D2S3C3 | 9.52 | 52.38 | 93.33 | 119.77 |
| D2S3C4 | 14.29 | 66.67 | 97.72 | 121.74 |
| D2S3C5 | 0.00 | 57.14 | 94.60 | 124.29 |
| D2S3C6 | 0.00 | 57.14 | 93.01 | 120.63 |
| D2S3C7 | 0.00 | 71.43 | 93.28 | 120.31 |
| D2S3C8 | 0.00 | 57.14 | 95.83 | 123.54 |
