# Supplementary material for: Exploring phenotypic diversity and constructing a floral core collection of Ziziphus jujuba var. spinosa
Source: PeerJ. 2026 Sep 24;14:e21708. doi: 10.7717/peerj.21708 (PMC13616194; doi:10.7717/peerj.21708)
Supplement: Supplementary file 10 [file peerj-14-21708-s011.docx]

| Sampling **Proportions**/% | MD/% | VD/% | CR/% | VR/% |
| --- | --- | --- | --- | --- |
| 10 | 28.57 | 57.14 | 91.70 | 130.66 |
| 15 | 9.52 | 71.43 | 94.42 | 131.17 |
| 20 | 14.29 | 71.43 | 95.58 | 126.46 |
| 25 | 9.52 | 71.43 | 98.59 | 123.84 |
| 30 | 9.52 | 66.67 | 98.62 | 119.98 |
| 35 | 4.76 | 57.14 | 98.88 | 116.55 |
| 40 | 0.00 | 52.38 | 99.38 | 115.32 |
