# Supplementary material for: Exploring phenotypic diversity and constructing a floral core collection of Ziziphus jujuba var. spinosa
Source: PeerJ. 2026 Sep 24;14:e21708. doi: 10.7717/peerj.21708 (PMC13616194; doi:10.7717/peerj.21708)
Supplement: Supplementary file 11 [file peerj-14-21708-s012.docx]

| Traits | Mean±SD | | CV (%) | | Variance | | *F*-test | *T*-test |
| --- | --- | --- | --- | --- | --- | --- | --- | --- |
|  | **Original Germplasm** | **Core Collection** | **Original Germplasm** | **Core Collection** | **Original Germplasm** | **Core Collection** |  |  |
| NOI | 10.61±1.49 | 10.59±1.78 | 14.00 | 16.81 | 2.21 | 3.17 | 0.13 | 0.91 |
| NOFI | 3.68±1.33 | 4.25±1.78 | 36.19 | 41.74 | 1.78 | 3.15 | 0.04 | 0.01 |
| NOF1 | 1.24±0.44 | 1.27±0.49 | 35.51 | 38.69 | 0.20 | 0.24 | 0.17 | 0.67 |
| NOF2 | 1.02±0.58 | 1.18±0.77 | 56.72 | 65.19 | 0.34 | 0.59 | 0.01 | 0.12 |
| NOF3 | 0.42±0.56 | 0.65±0.66 | 132.06 | 101.69 | 0.31 | 0.43 | 0.07 | 0.02 |
| PEL | 3.14±0.73 | 3.22±0.93 | 23.19 | 28.85 | 0.53 | 0.86 | 0.03 | 0.49 |
| FD | 5.82±0.57 | 5.97±0.73 | 10.74 | 12.21 | 0.32 | 0.53 | 0.00 | 0.11 |
| FDD | 2.45±0.28 | 2.52±0.38 | 11.59 | 15.00 | 0.08 | 0.14 | 0.00 | 0.14 |
| CL | 1.80±0.17 | 1.82±0.21 | 9.49 | 11.57 | 0.03 | 0.04 | 0.03 | 0.38 |
| CW | 1.65±0.18 | 1.69±0.22 | 10.92 | 12.92 | 0.03 | 0.05 | 0.04 | 0.23 |
| CAR | 1.09±0.08 | 1.08±0.09 | 7.22 | 8.07 | 0.01 | 0.01 | 0.33 | 0.55 |
| PL | 1.57±0.25 | 1.65±0.40 | 15.75 | 23.96 | 0.06 | 0.16 | 0.01 | 0.08 |
| PW | 0.91±0.15 | 0.95±0.21 | 17.06 | 22.23 | 0.02 | 0.04 | 0.03 | 0.11 |
| PAR | 1.09±0.29 | 1.08±0.09 | 7.22 | 8.07 | 0.01 | 0.01 | 0.33 | 0.55 |
| SL | 1.62±0.18 | 1.65±0.25 | 11.27 | 14.87 | 0.03 | 0.06 | 0.01 | 0.22 |
| FL | 1.08±0.16 | 1.12±0.21 | 14.49 | 18.93 | 0.02 | 0.05 | 0.00 | 0.09 |
| PIL | 0.80±0.16 | 0.81±0.21 | 20.39 | 25.32 | 0.03 | 0.04 | 0.04 | 0.58 |
| FDT | 0.26±0.10 | 0.30±0.18 | 38.04 | 60.16 | 0.01 | 0.03 | 0.00 | 0.05 |
| FDA | 4.81±1.20 | 5.24±1.68 | 25.05 | 32.05 | 1.45 | 2.82 | 0.00 | 0.04 |
| FA | 19.32±3.37 | 20.19±4.58 | 17.42 | 22.69 | 11.33 | 20.99 | 0.00 | 0.12 |
| FDP | 0.25±0.04 | 0.26±0.06 | 17.08 | 23.45 | 0.00 | 0.00 | 0.11 | 0.18 |
